# Long-Term Memory Dysfunction in Limbic Encephalitis

**DOI:** 10.3389/fneur.2019.00330

**Published:** 2019-04-26

**Authors:** Niels Hansen

**Affiliations:** ^1^Department of Epileptology, University of Bonn Medical Center, Bonn, Germany; ^2^Department of Psychiatry and Psychotherapy, University of Goettingen, Goettingen, Germany

**Keywords:** autoimmunity, limbic encephalitis, long-term memory, autoantibodies, synaptic consolidation

Limbic encephalitis (LE) is an autoimmune disease defined by clinical criteria, such as seizures, psychiatric and in particular working memory abnormalities in conjunction with apparative criteria underlying structural or functional changes in the temporal lobe according to autoimmune encephalitis guidelines (1). Working memory encompasses a transient encoding of information in readiness for further processing within a time window of seconds during cognitive task operations based on neurophysiological mechanisms, such as short-term synaptic facilitation (2). On the contrary, long-term memory (LTM) serves to encode, consolidate, and finally store information for long intervals ranging from minutes to months or even life (3) through cellular mechanisms, such as long-term potentiation (LTP) (4).

In translational transfer experiments of autoimmune encephalitis from humans to the mouse, critical impairment of synaptic LTP in the hippocampus was proved by autoantibodies against the N-methyl-D-aspartate receptor (NMDAR) (5) and alpha-amino-3-hydroxy-5-methyl-4-isoxazolepropionic acid (AMPA) receptor subunit GluA2 (6). Synaptic LTP in the hippocampus is considered to be a mechanism of synaptic consolidation (7) serving to enable LTM storage within the hippocampus. Not only antibodies against glutamatergic receptors cause LTM dysfunction probably due to altered synaptic transmission and plasticity—LGI1-LE is also assumed to be based on a synaptic mechanism, as hippocampal AMPA 2 receptors are reduced via neutralization of LGI1-ADAM22 interaction by LGI1-antibodies (8). AMPA receptor reduction is functionally partially equivalent to enhanced AMPAR endocytosis as fewer AMPARs become available for synaptic transmission. LTP might in turn be impaired (9) and LTM formation as well consequently via this suggested process.

On the contrary, the pathophysiology of accelerated long-term forgetting (ALF) is still not well-understood. It occurs often in temporal lobe epilepsy patients (10), has been reported recently in LE, and is even more predominant in LE not associated with autoantibodies (11) (Table 1A). ALF can be assessed by neuropsychological tests assessing long-term free recall entailing a 1-week time period with not derogated free recall after half an hour (11). ALF is believed to be based on a shortage of memory consolidation (23), so that ALF in LE clearly depicts LTM dysfunction. Persistent deficits in memory retrieval (19) and recognition (14), anterograde (21) and retrograde (24), autobiographic (25), visuospatial, verbal, and episodic LTM deficits have been reported in LE patients (15, 19, 20, 22, 26, 27) (for reports on LTM dysfunction in adult LE patients see Table 1A, *n* ≥ 6). These distinct facets of LTM deficits often occurred in LE patients in association with antibodies against membrane surface antigens, such as voltage-gated potassium channels (VGKC) with its subgroups of leucine-rich glioma-inactivated 1 (LGI1) and contactin-associated protein-like 2 (CASPR2), AMPARs as well as antibodies against intracellular antigens, such as glutamic acid decarboxylase 65 (GAD65) and Ma/Ta2. The time frame of LTM decline ranged from a month to years (15, 16, 19, 22, 28) and even decades, with dynamic fluctuations in memory capacity over time (18) (Table 1A).

**Table 1A:** Reports in limbic encephalitis patients on memory including LTM dysfunction.

**Antibodies**	**No. affected/all patients (*n* ≥ 6)**	**Patient's age mean ± sd or median (range)**	**Neuropsychology tests**	**Neuropsychology results**	**MRI**	**References**
Abs–	24/28	48 ± 15	DCSR, VMLT	Impaired verbal and figural memory	Mesiotemporal signal changes	(12)
Abs–, Abs+	23	44 (17)	VLMTe, RAVLT, DCSR	ALF in 31–67%	5% AHS, AE in 48%	(11)
Abs–	20–22/40	51 (10-73)	DCSR, VLMT	Verbal or figural deficit	Mesiotemporal signal changes	(13)
Abs–	1/6	–	CVLT	Anterograde amnesia, impaired retrieval and recognition		(14)
CASPR2	1/6	–	CVLT	Impaired retrieval and recognition	–	(14)
GAD65	16	–	VLMT, DCSR	Verbal and figural memory deficit	Mesiotemporal signal changes	(15)
GAD65	11	43.1 ± 11	VLMT, DCSR	45% verbal, 64% figural memory impairment	100% hippocampus and 73% amygdala affection	(16)
GAD65	7/12	30 (16-48)	VLMT, DCSR	Verbal and figural memory deficit	Greater amygdala volume within 12 months of disease vs. control	(17)
GAD65	5/7	35.3 ± 10.2	VLMT, DCSR	2/5 impaired verbal and figural memory	Signal hyperintensity in hippocampus in 2/5, atrophy of hippocampus in 3/5	(18)
GAD65	1/6	–	CVLT	Impaired retrieval and recognition	–	(14)
LGI1	30	66 ± 13	RAVLT, ROCF	Impairment of delayed recall	CA2/3, DG atrophy, reduced microstructure integrity	(19)
LGI1	27	66 ± 11	RAVLT, ROCF	Verbal and visual episodic memory impairment	Hippocampal hyperintensities 30% unilateral, 48% bilateral	(20)
Ma/Ta2	1/7	35.3 ± 10.2	VLMT, DCSR	Impaired verbal and figural memory	Unilateral amygdala enlargement, unilateral amygdala and hippocampus atrophy	(18)
Ma/Ta2	1/6	–	CVLT	Anterograde amnesia, impaired retrieval memory and recognition	–	(14)
NMDA, VGKC	1/7	35.3 ± 10.2	VLMT, DCSR	Impaired verbal and figural LTM	Hippocampal atrophy, amygdala enlargement	(18)
Paraneoplastic	11	41.5 ± 13.2	VLMT, DCSR	73% verbal and 64% figural memory impairment	64% hippocampus and 73% amygdala affection	(16)
VGKC	19	60.1 ± 15	WMS III	Impaired memory recall	Hyperintensities in temporal lobe	(21)
VGKC	15/18	55 (20-73)	VLMT, DCSR	Verbal memory impairment	Mesiotemporal signal changes 68% bilateral, 28% left, 11% right sided	(22)
VGKC	15/15	–	VLMT, DCSR	Verbal and figural memory deficit	Mesiotemporal signal changes	(15)
VGKC	12–14/15	59.9 (19-72)	VLMT, DCSR	Verbal or figural memory deficit	Larger volumes of amygdala and hippocampus within 12 months of disease vs. control	(17)
VGKC	11/18	55 (20-73)	VLMT, DCSR	Figural memory impairment	Mesiotemporal signal changes 68% bilateral, 28% left, 11% right	(22)

*Abs–, no antibodies; Abs+, antibodies; AE, amygdala enlargement; AI, autobiographic interview; ALF, accelerated long-term forgetting, AM, autobiographical memory testing; AMPA, alpha-amino-3-hydroxy-5-methyl-4-isoxazolepropionic acid; CASPR2, contactin-associated protein-like 2; CVLT, California verbal learning test; DCSR, Diagnosticum für Cerebralschädigung revised; GAD65, glutamic acid decarboxylase 65; LTM, long-term memory; MRI, magnetic resonance imaging; MTL, medial temporal lobe; NMDA, N-methyl-D-aspartate; RAVLT, Rey Auditory Verbal Learning Test; ROCF, Rey-Osterrieth Complex Figure Test, VGKC, voltage gated potassium channels; VLMTe, Verbaler Lern und Merkfähigkeitstest extended version; VMLT, Verbaler Lern und Merkfähigkeitstest; WMS III, Wechsler Memory scale III*.

Functional memory impairment seems to be based on the structural integrity of mesiotemporal brain structures, as memory function is known to correlate with reduced hippocampal subfield volumes, e.g., the CA1-4, dentate gyrus or subiculum in VGKC or paraneoplastic antibody positive-encephalitis (16, 29). LE involves frontal lobe structures (20) also, but the main underlying brain pathology on the macroscopic (17) and microscopic level (involving infiltrating lymphocytes) affects the amygdalohippocampal complex (30) indicating that dysfunctional LTM is more probable than impaired working-memory pathways. In particular, some LE forms are susceptible to LTM dysfunction as their disease-mediating antibodies against membrane receptors, such as AMPA- (31), NMDA- (32), metabotropic glutamate receptor 5 (mGluR5) receptors (33) are critically involved in hippocampal synaptic long-term plasticity and LTM formation (9, 34, 35). It is thus not surprising that some AMPAR-antibodies associated LE patients present with an amnestic syndrome, such as the unique clinical manifestation of autoimmunity (36). Antibody-mediated immunopathology involving distinct memory phenotypes fluctuates (18), but its pathogenic antigen-antibody interaction of glutamatergic receptors often take days to develop functional changes in receptor electrophysiology [neuronal incubation with antibody serum requires days: Ohkawa et al. (8)] and antibody-directed epitopes undergo post-translational changes in protein expression (37), indicating time preconditions to worsen LTM function. LTM dysfunction is not a unique feature of limbic dysfunction induced by autoimmunity, but can also be caused by viral encephalitis, such as herpes simplex encephalitis. The clinical features of viral encephalitis affecting the temporal lobe can resemble those of autoimmune-mediated limbic encephalitis (LE), but frequently start with a more fulminant onset, often with fever or aphasia. The diagnosis of viral encephalitis must be ascertained by detecting viral DNA in the cerebrospinal fluid via a polymerase chain reaction. The type of LTM impairment in herpes simplex encephalitis affecting either verbal memory (pattern a) and/or memory of names (pattern b) (38), and/or memory of living things (pattern c) (39) depends on structural lesions in the temporal lobe [involving the hippocampus (a) (38) or the lateral temporal lobe (b) (38) or antero-medial temporal cortex (c) (39)].

The occurrence of episodic LTM deficits in LE are often not followed by working memory disturbances (14), so that there may be patients suffering from LE who are not registered due to application of the Graus et al. criteria (1). Redefining the memory criteria in LE has been proposed to consider episodic LTM function in LE patients (14). We suggest an even more amplified and elaborated LTM-dysfunction criterion in addition to working memory performance to adapt LE criteria to include several aspects of episodic, semantic and visuospatial LTM and ALF. Thus, to diagnose limbic encephalitis, we suggest incorporating this aforementioned novel memory criterion within the existing criteria from Graus et al. (1) (Table 1B). Furthermore, we recommend utilizing specific neuropsychological tests (Table 1C) to detect subtle LTM deficits in LE patients.

**Table 1B:** Proposal for revised criteria for limbic encephalitis.

We suggest using a novel memory criterion to diagnose limbic encephalitis.
The first criterion in both potential and definitive autoimmune encephalitis in the Graus criteria (1) should be amplified by specifying the term “working memory deficits” by “short- and/or long-term memory deficits.”

**Table 1C:** Neuropsychological test battery for assessing LTM function in limbic encephalitis.

1. Figural/visuo-spatial learning and memory including long-term memory via the “Diagnosticum für Cerebralschädigung” (DCS-R) (40)
2. Visuocontruction via the “Rey-Osterieth Complex Figure Test”
3. Verbal memory including long-term memory and accelerated long-term forgetting via the “Extended version of the Verbal Learning and Memory Test” (extended version VLMT) (11)

This suggested framework provides a more realistic imprint of memory impairment in LE and might help us identify and treat LE patients with LTM disabilities. This is particularly important, as early immunosuppressive or other treatment options (e.g., tumor resection) are essential to improve or recover memory performance in LE patients.

## Author Contributions

The author confirms being the sole contributor of this work and has approved it for publication.

### Conflict of Interest Statement

The author declares that the research was conducted in the absence of any commercial or financial relationships that could be construed as a potential conflict of interest.
